# Mouse models and human islet transplantation sites for intravital imaging

**DOI:** 10.3389/fendo.2022.992540

**Published:** 2022-10-05

**Authors:** Leslie E. Wagner, Olha Melnyk, Bryce E. Duffett, Amelia K. Linnemann

**Affiliations:** ^1^ Department of Biochemistry and Molecular Biology, Indiana University School of Medicine, Indianapolis, IN, United States; ^2^ Department of Pediatrics, Indiana University School of Medicine, Indianapolis, IN, United States; ^3^ Center for Diabetes and Metabolic Diseases, Indiana University School of Medicine, Indianapolis, IN, United States

**Keywords:** diabetes, human islets, intravital microscopy, islet transplantation, humanized mice

## Abstract

Human islet transplantations into rodent models are an essential tool to aid in the development and testing of islet and cellular-based therapies for diabetes prevention and treatment. Through the ability to evaluate human islets in an *in vivo* setting, these studies allow for experimental approaches to answer questions surrounding normal and disease pathophysiology that cannot be answered using other *in vitro* and *in vivo* techniques alone. Intravital microscopy enables imaging of tissues in living organisms with dynamic temporal resolution and can be employed to measure biological processes in transplanted human islets revealing how experimental variables can influence engraftment, and transplant survival and function. A key consideration in experimental design for transplant imaging is the surgical placement site, which is guided by the presence of vasculature to aid in functional engraftment of the islets and promote their survival. Here, we review transplantation sites and mouse models used to study beta cell biology *in vivo* using intravital microscopy and we highlight fundamental observations made possible using this methodology.

## Introduction

Diabetes is a growing public health concern, with an estimated 536.6 million people currently diagnosed worldwide and a projected increased incidence to 783.2 million people by 2045 (1). Both type 1 and type 2 diabetes are characterized by failure of beta cells within the islets of Langerhans of the pancreas, resulting in defective insulin secretion from the beta cells that leads to clinical hyperglycemia. Although significant progress has been made in treatments for both type 1 and type 2 diabetes, we do not yet have a full picture of the pathophysiology of either disease. In fact, we, as a field, have only recently begun to fully understand what constitutes a normal pancreatic islet (2). This is due, at least in part, to caveats with the models used to study diabetes and in particular the nature of how islets are often examined in isolation. For the study of human islets, we are limited by the relative inaccessibility of the pancreas as well as obvious ethical issues that require evaluation of islets only after their removal from cadaveric donors. Although evaluation of isolated islets has provided a wealth of valuable fundamental information, this approach makes it difficult to answer questions that are intrinsic to the *in vivo* islet niche. Proper functioning of endocrine cells within the islets requires crosstalk with numerous cell types found in the local microenvironment, including vascular cells, neuronal cells, immune cells, and the exocrine pancreas (3).

Therefore, models to study islet function and dysfunction should take the islet niche, and connections with other cell types beyond the islet, into consideration to allow for physiologically relevant evaluation of both function and dysfunction. One way to mimic the local islet microenvironment is to transplant the isolated islets into a rodent to provide an environment containing some of the same cell types of the pancreatic niche. Importantly, when using islets isolated from human donors, an immunodeficient or humanized mouse model must be used to prevent rejection. While neither of these models allow for perfect recreation of the local islet microenvironment, both can be used as powerful tools to study beta cell biology, allowing for interaction with the local vasculature and effective engraftment. Humanized mouse models also allow incorporation of immune cell-islet interactions by attempting to replicate a host immune system (4). Although not yet shown with transplanted human islets, reinnervation has been demonstrated with mouse islets allografts transplanted under the kidney capsule of inbred wild type mice (5). Thus, current models used to transplant islets to study beta cell biology can recapitulate many cell-cell interactions found in the local islet microenvironment, and future developments are likely to continue to increase capabilities.

**Figure 1:**
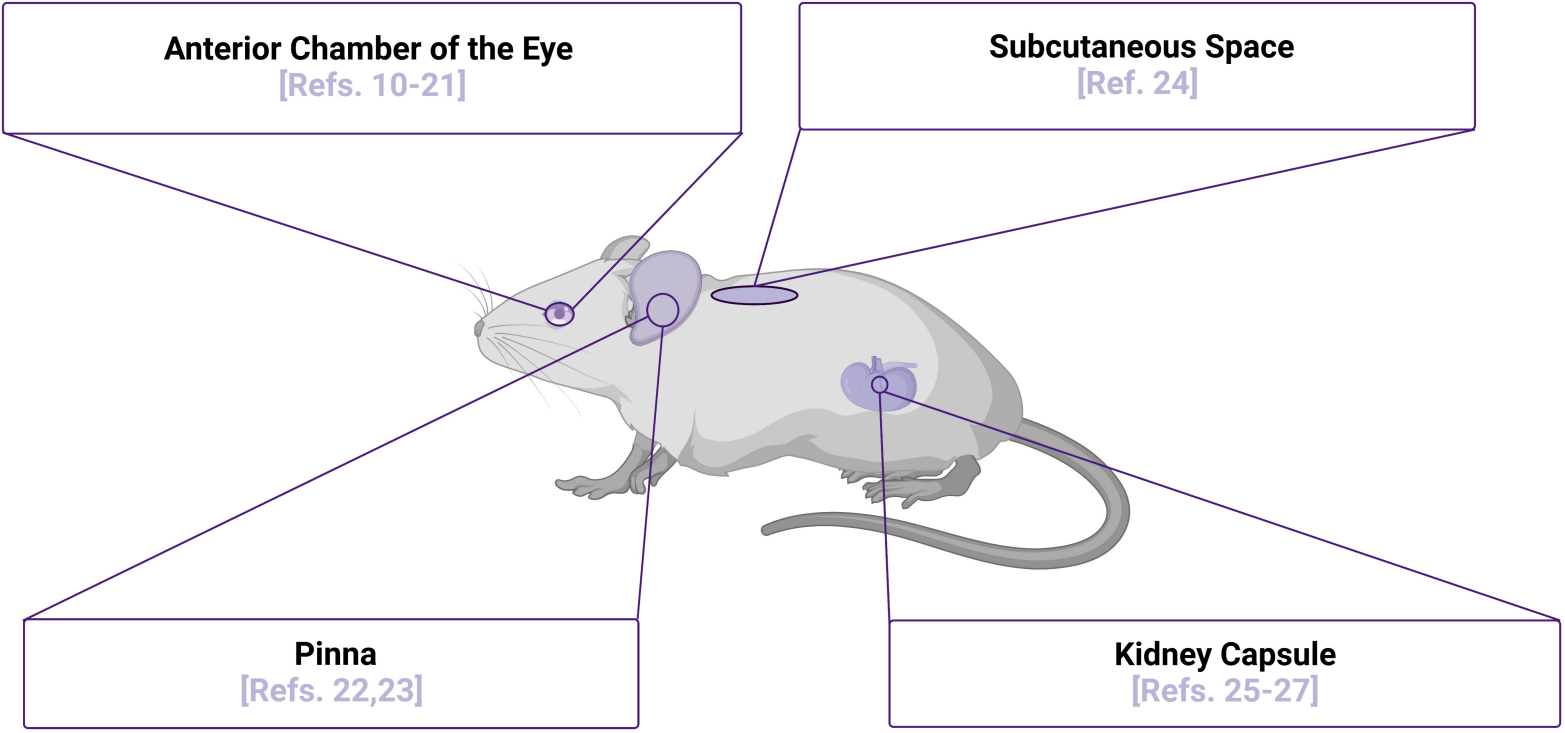
Islet Transplantation Sites for Intravital Microscopy Purposes. Multiple sites of islet transplantation may be used to perform intravital imaging studies to elucidate mechanisms of islet biology, several of which are highlighted along with relevant references numbered. Although the pinna of the ear has not been used as a transplantation site to image human islets *in vivo* to date, there is potential for this site with several studies demonstrating that the xenografts are functional and can be readily imaged. Image was created using BioRender.com.

The initial engraftment of islets is a highly stressful period when islet viability is challenged and limited by multiple factors that can vary depending on site of transplantation, with one major factor being revascularization of the xenografts. Vascular cells have been shown to play a significant role in regulation and support of several physiological processes in beta cells, including proliferation, differentiation, insulin secretion, and viability (6–9). With vasculature being a major factor in determining proper islet engraftment, survival, and function, highly vascularized areas such as the kidney capsule and the anterior chamber of the eye (ACE) provide promising sites for transplantation of islets, as highlighted in **Figure 1**. Also highlighted in **Figure 1** are the pinna of the ear and the subcutaneous space, sites that are not well-vascularized innately, but may be induced to vascularize islet xenografts. In the sections below, we will highlight studies of human islets in each of these transplant sites, with a particular focus on the use of intravital microscopy as an approach to study their function. Intravital microscopy is a method in which conventional confocal, widefield fluorescence, or multiphoton microscopy are employed to collect images within living organisms, providing comprehensive insight into dynamic processes occurring within the body (28–32). Since first introduced by Antony van Leeuwenhoek to image nearly translucent tissues with bright-field transillumination (33), the technique of intravital microscopy has progressed significantly with advances in microscope technology. This technological advancement has occurred in parallel with expansion of experimental imaging techniques, significantly increasing a researcher’s capability to study complex biological interactions over time. Of note, the development of both optical fiber implantation (34) and imaging windows have allowed for longitudinal monitoring of dynamic physiological processes *in vivo*. Prior to these advancements, intravital microscopy was only possible for a single session due to requirement of terminal surgeries. The ability to longitudinally image a physiological event *in vivo* was first introduced seven decades ago with use of the dorsal skin window chamber model, allowing angiogenesis to be observed in real time (35). To date, intravital imaging utilizing these longitudinal techniques has now been used to study an array of tissue types and organ systems, including the skin, brain (36), mammary gland (37), lung (38), pancreas (25), kidney (39), liver (40), lymph nodes (41), bones such as the femur (42), and embryos (43–46). Although these studies were imperative to push the field of intravital microscopy forward, in this review, will focus solely on intravital microscopy studies of transplanted islets. We will also highlight how these approaches have improved our understanding of human islet biology and discuss how technological advancements continue to move the field forward in this realm.

## Intravital microscopy as a tool to study transplanted islets

To study islet physiology and alterations in function during disease pathogenesis and stress response, intravital microscopy has emerged as a powerful tool for discovery, containing different microscope options to optimize user experience. In combination with fluorescent probes, widefield fluorescence, confocal or multiphoton microscopy are the most used options for intravital microscopy (47–49). Widefield fluorescence microscopy uses a powerful light source (mercury or xenon lamp) to illuminate fluorophores within living tissue, potentially causing tissue damage during prolongated imaging. However, despite this potential drawback, big area overviews can be imaged simultaneously at relatively high speeds, with detailed structural morphologies visualized. Currently, no work has been published utilizing widefield fluorescent microscopy of engrafted islets due to limitations of this approach and low system availability. Confocal microscopy utilizes lasers with a pinhole technology as a light source, illuminating highly defined areas of interest within a tissue. This technology allows for an increase in spatial resolution and reconstructed 3D views of the objects but has limitations in the laser light penetration depth and potentially causes phototoxic effects during prolonged imaging sessions. Using an excitation wavelength in the infrared spectrum, a multiphoton system can penetrate deeper into a tissue of interest, achieving depths of over several hundred μm (31), thus providing unprecedented access to tissues in the context of live animal imaging. Also unique to multiphoton imaging systems, structures such as fibrillar collagen, myosin, and myelin may be visualized without addition of any exogenous probes due to its unique second harmonic generation capabilities (50, 51). Third harmonic generation is also possible due to the high-power output of infrared pulse lasers, and this technique allows visualization of lipid droplets as well as the species discussed for second harmonic generation. The combination of these unique techniques with conventional fluorophores increases the potential observation parameters to study with multiphoton microscopy. Intravital microscopy can be used to study many physiological events in live animals, allowing researchers to detect changes in complex biological processes as they occur in the organism and has been used to study many aspects of islet biology, including immune interactions in diabetes pathogenesis (52–55), islet vasculature morphology and flow rate (56), and changes in redox state and calcium dynamics (25). However, with the rapid pace of advancement in microscope technology, image analysis software, fluorescent probes, and biosensors, there are continuously evolving biological applications and potential for discovery using this approach.

## Transplantation sites for fluorescent imaging purposes

### Kidney capsule

In murine studies, the most common transplant site for human islets is beneath the kidney capsule (57). The kidney capsule provides a smooth surface that is beneficial for imaging, and the islets become well vascularized (26, 58), provided with the necessary nutrients to thrive and function. In the endogenous pancreas, a well vascularized environment also allows for efficient dissemination of insulin released from the islets (10), with numerous studies demonstrating transplant restoration of euglycemia in several diabetic immunodeficient mouse models (58–60). With sufficient vascularization, transplanted islet grafts are stable and can survive for over a month within the host animals (61, 62), with the current longest reported time of survival being over 300 days (60).

**Figure 2:**
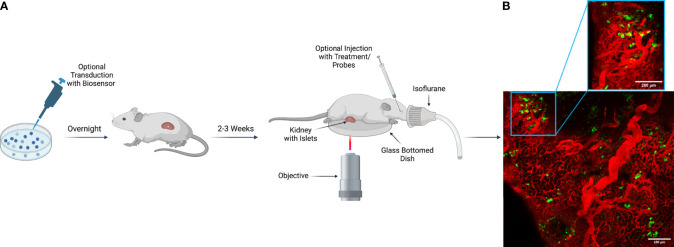
Imaging Scheme for Intravital Imaging of Transplanted Human Islets Under the Kidney Capsule. **(A)** Human islets isolated from a cadaveric donor can be transduced with a virally packaged biosensor as desired to visualize function *in vivo*. Transduced islets then remain in viral media overnight to gain optimal biosensor expression before transplantation into a recipient animal the following day. If the experimental design does not include transduction, islets can be transplanted after overnight recovery from shipment. Following delivery of the islets to the recipient, a 2–3-week period is required for islet engraftment and recovery. During this time, the islets become optimally vascularized, and transduced biosensors mature. For terminal imaging experiments, the recipient animal is anesthetized, and the kidney is externalized and sutured for stable positioning during image acquisition. After externalization, the kidney is placed upon a glass bottom dish, and the animal is transferred to the microscope stage. Once upon the stage, various treatments, fluorescent probes, and antibodies can be injected for evaluation of islet physiology and cellular response to stimuli. Image was created using Biorender.com. **(B)** Representative example of human islets engrafted into the kidney capsule of an NSG mouse. In both the tiled image of a large region of the pancreas, as well as in the zoomed in view, islet beta cells can be observed expressing a ratiometric biosensor to measure reactive oxygen species under the control of a rat insulin promoter [Ins-GRX1-roGFP2 (25)] with vasculature marked by IV-injected albumin conjugated to AlexaFluor-647.

After successful transplantation, intravital microscopy can be employed as a tool to measure beta cell biology and disease pathophysiology *in vivo*. To study specific mechanisms of islet physiology, various virally packaged biosensors have been used successfully to study islet and beta cell responses to stimuli. With transplanted islets, a biosensor of interest can be used to transduce islets *in vitro* prior to transplant into the animal (25, 27). Following transplantation, we have found that within 2-3 weeks, islets become properly vascularized and gain optimal biosensor expression (25). To perform terminal intravital microscopy on islets transplanted under the kidney capsule, live animals under anesthesia require only a minimally invasive surgery to externalize the kidney for imaging. While placed upon the microscope stage, various treatments, antibodies, and fluorescent probes can then be introduced into the animal utilizing multiple routes of administration that include retro-orbital, intraperitoneal, and subcutaneous injection (**Figure 2A**). This highlights the importance of a well-vascularized environment surrounding the transplanted islets, because without sufficient vascularization, not only will the graft undergo cell death in the immediate post-transplantation period, but the injected reagents would also be unable to reach their target within the transplanted islets.

Abdominal imaging windows may also be surgically implanted for intravital imaging, allowing longitudinal monitoring of biological processes in human islets transplanted under the kidney capsule (26). The abdominal imaging windows are typically constructed of the glass coverslip supported by the titanium frame, which is surgically inserted and sutured on top of the organ of interest (63). However, this conventional window construct is limited due to the rigidity of the materials, which can lead to window detachment and further complications of skin irritation, tissue degradation, and inflammatory responses. To address those limitations, flexible imaging windows, previously used to image organs such as the pancreas, open a new avenue for the longitudinal intravital imaging of transplanted islets under the kidney capsule of mice (64).

Islets transplanted under the kidney capsule are fully functional and biological response to stressors can be monitored. Lentivirus containing human insulin promoter–GFP (HIP-GFP) has been employed to aid in the marking of transplanted human islets under the kidney capsule, allowing researchers to inject Texas Red-conjugate dextran to visualize vascularization following engraftment (65). With respect to function, we have found that transplanted mouse islets expressing a beta cell selective GRX1-roGFP2 biosensor to detect reactive oxygen species (ROS) respond rapidly to systemic administration of the toxic glucose analog and ROS-generator, alloxan (25). Similar to mouse islets, human islets from cadaveric organ donors are also well engrafted and express GRX1-roGFP2 sensor robustly two weeks after transplant surgery (**Figure 2B**). Though no studies currently exist evaluating human islet function in the kidney capsule using genetically encoded fluorescent biosensors, the components are in place for future discovery in this context.

### Anterior chamber of the eye

Just over ten years ago, groundbreaking research identified the anterior chamber of the eye (ACE) as a site for successful islet transplantation and this discovery has progressed into clinical trials in T1D patients (66, 67). Indeed, the eye is widely used as a transplantation site for longitudinal islet studies (68, 69). The ACE is an oxygen-rich and metabolic stress reducing environment that has also been described as an immune-privileged environment (65, 70), meaning that allografts may resist rejection through limited immune cell migration from systemic circulation *via* the blood-ocular barrier (71, 72). However, this has been challenged by observations of rejection (73, 74) and visualization of immune attack (75) when transplanting islets in the ACE. After transplantation, islets readily engraft on the iris, a highly innervated and vascularized structure. Using the ACE as a transplant site allows for less invasive longitudinal imaging than in other sites, utilizing the eye as a natural imaging window (11). Animals undergo a minimally invasive surgery to transplant the islets into the ACE and recipient animals recover quickly, permitting single-cell resolution imaging within days after transplantation. Repeated imaging of islets transplanted into the ACE also does not require additional surgery or maintenance of an imaging window, making it an appealing method for *in vivo* imaging of transplanted islets. Both human and murine islets transplanted into the ACE become well vascularized (12, 13), allowing for physiological interaction with vascular endothelial cells and pericytes as in the native microenvironment. In murine islets, angiogenesis begins roughly one day after transplantation (14), but vascular density similar to what is seen in the native islet environment is not observed until roughly four weeks following delivery (76). Studies have also shown that the highly innervated iris provides a supply of sympathetic and parasympathetic nerves to transplanted mouse islets (15), with no studies to date showing this result with human islets. To ensure that the surgical procedure does not lead to sustained interocular pressure (IOP) within the eye, researchers have utilized contact lenses equipped with a strain sensor to measure IOP and found that it was slightly elevated after the procedure, but this mild increase was diminished two weeks following islet transplantation (16). This result is reassuring, suggesting that the surgery does not cause lasting damage to the recipient due to buildup of ocular pressure.

Many imaging experiments to study beta cell biology have been performed using transplanted human islets in the ACE, including the study of NAD(P)H response (12), calcium dynamics (17), and beta cell mass (18). Much as with the transplantation of islets under the kidney capsule, biosensors can be utilized to infect islets prior to transplantation to report upon islet responses to stimuli (19). For example, an adenoviral calcium biosensor, GCaMP6m, has been used to infect human islets transplanted into the ACE of immunocompromised mice, demonstrating that islet calcium dynamics were functionally conserved (20). Also, once the animal is being imaged, injection of various treatments and probes may be employed to study beta cell biology during imaging, such as injection of a vasculature dye or cell death marker (21). Aside from using islets isolated from cadaveric donors, human induced pluripotent stem cells (hiPSCs) can be differentiated *in vitro* into islet-like structures for transplantation purposes, potentially reducing the burden to obtain human tissue for future studies (21), making exciting future discoveries using these cells possible.

### Pinna

Advances in ear reconstructive surgery to correct congenital malformation or injury highlight the capacity for this tissue to support remodeling and acceptance of recipient tissue (77, 78). To date, two research groups have performed pioneering studies of murine islets transplanted into the pinna of the ear, a site that is advantageous for the potential of performing non-invasive intravital imaging. In one instance, the islets were transplanted into the pinna without any additional supportive matrices and insulin positive cells persisted at 12 weeks post transplantation as detected by immunofluorescent staining (22). In this study, multiphoton intravital microscopy resolved fluorescent T-cell infiltration within the transplanted islets, demonstrating that this transplantation site is indeed suitable for non-invasive *in vivo* imaging studies (22). Of note, in other studies when islets were again transplanted with no additional matrices, the islets were not able to restore STZ-induced hyperglycemia (23). This suggested a defect in microenvironment of the islets that impacted stimulus/secretion coupling or engraftment and precluded optimal function. However, when this group co-transplanted the islets with epididymal fat pad, basement membrane matrix, rat tail collagen and basic fibroblast growth factor or rat tail collagen and vascular endothelial growth factor, they found that hyperglycemia was reversed (23). Subsequent intravital imaging on transplanted islets expressing GFP confirmed that this transplantation site can be used for intravital microscopy studies.

### Subcutaneous space

Another minimally invasive and easily accessible area of islet transplantation is the subcutaneous space. Although subcutaneous islet transplant studies commonly involve the study of euglycemia restoration following diabetes induction, this method of transplantation has also been utilized in intravital microscopy studies involving photoacoustic imaging due to proximity to surface (24). However, to date, this method of imaging has only been used to study transplant revascularization in syngeneic recipient animals (24). The major drawback of using this site for islet transplantation is the relative avascularity of this environment compared to other transplantation sites discussed above. Thus, an additional method must first be deployed to ensure engraftment, such as the induction of an immune response driving angiogenesis with insertion of a hollow nylon catheter (24) or through the use of a viability matrix with transplantation (23). Additionally, co-transplantation of islets and adipose-derived microvessels into the subcutaneous space may be employed to accelerate the establishment of a host-graft vascular network (79). When the initial hypoxic graft response is reduced, an environment mimicking native islet vascular conditions can be maintained. The subcutaneous space is thus of significant interest for its ease of access, and studies of human islets alone (79) or in islet containment devices (80) for studies focused on therapeutic islet replacement have produced promising and exciting results in recent years.

## Mouse models used for transplantation

Several mouse models can be employed as experimental hosts in the study of human islet transplantation, including both immunodeficient and humanized strains of mice. While immunodeficient mice are selected as hosts for transplantation to prevent rejection of an islet xenograft, studies of human islets and their interaction with the immune system can be performed using humanized mice models.

### Immunodeficient mice

To transplant human islets into mice, an immunodeficient mouse model must be used to prevent xenograft rejection. The most common immunodeficient mouse model for islet xenografts is the NOD.Cg-*Prkdc^scid^Il2rg^tm1Wjl^* mouse model, also known as NOD-*scid* gamma (NSG) mouse strain. In this severely immunodeficient model, the *Il2rg*, or the IL-2 receptor gene, is knocked out completely, resulting in a lack of mature natural killer (NK) cells. Also, the severe combined immunodeficiency, or *scid*, mutation is present in *Pkrdc*, a DNA repair protein. With this mutation, there is a severe combined immune depletion of both B and T cells, hence the *scid* designation. Several studies have used this strain for human islet transplants without rejection reported (26, 59, 60, 81). However, for longer term studies, graft *vs* host disease (GVHD) is still a concern. MHC class I/II null mice have been developed to address this (82) and are now commercially available from Jackson Labs.

Several additional models with various degrees of immune depletion have also been used for human islet transplant studies. This includes CB17.Cg-*Prkdc^scid^Lyst^bg-J^*/Crl (SCID beige) mice, a congenic line that results in defective NK cells, T cells, and B cells through mutations in *Pkrdc* and *Lysbtg* (79, 83, 84). Another immunocompromised mouse model occasionally used in human islet transplants is the CAnN.Cg-*Foxn1^nu^*/Crl (BALB/c nu/nu) mouse model (85, 86). This mouse lacks a functional thymus, rendering the animals incapable of forming functional T cells. Like the BALB/c Nude mice, the outbred Crl : NU*-Foxn1^nu^* (NU/NU) mouse has also been used for human islet transplantation experiments (20, 87). Originally believed to be a congenic BALB/c line, this mouse model is outbred and lacks a functional thymus, leading to T cell deficiency.

The NOD-*Rag1^null^ IL2r*γ*^null^ Ins2* ^+/^ *Akita*, or NRG-Akita, strain is uniquely suited to studies of xenograft performance in the absence of an endogenous complement of beta cells to maintain glycemia. These mice develop spontaneous hyperglycemia due to a mutation in the insulin 2 gene that leads to improper proinsulin folding and severe ER stress-induced beta cell death. These mice are rendered immunodeficient by null mutations of *Rag1* and perforin 1 (*Prf1*) genes, leading to inactivated NK cells and immature B cells and T cells. Several studies have used this line for human islet transplants since it prevents transplant rejection while maintaining the disease state of hyperglycemia through ablation of endogenous beta cells (79, 88). Under these conditions, the transplant bears the full burden of insulin secretion to obtain normoglycemia. While this effect can be obtained in other models by endogenous beta cell ablation using the toxic glucose analogue, streptozotocin (STZ), prior to islet transplant, some researchers may prefer the simplicity of endogenous beta cell death without the necessity of the use of a biohazard such as STZ.

### Humanized mice

Although mice provide many valuable insights into biological processes, many findings from studies in mice do not translate well into clinical settings. To study aspects of human biology *in vivo*, humanized mice were created, allowing the study of the human immune system in several disease contexts, such as autoimmune disorders, cancer, and infectious diseases. Humanized mice are defined as immunodeficient mouse models that are engrafted with peripheral blood mononuclear cells (PBMCs) or hematopoietic stem cells, allowing for study of the human immune response in certain contexts, such as immune interaction with transplanted islets (4). PBMCs are comprised of multiple types of immune cells such as T cells, B cells, NK cells, and monocytes. While commonly used to study mechanisms of xenograft rejection and immunosuppressive agents as preclinical therapeutics (89–92), these models can readily be used to study interactions between human immune cells and transplanted human islets *in vivo* with intravital microscopy. When utilizing these models to perform intravital imaging, several aspects of the immune response can be studied, providing mechanistic insight on how the human immune system reacts to allogenic transplants. Although studies have not been performed using intravital microscopy of transplanted human islets in humanized mice to date, several studies have been conducted using intravital imaging of humanized mice in different contexts (93, 94), showing that these mouse models may be used for human islet intravital imaging experiments in the future. As the race to develop cell-based insulin replacement therapies continues, intravital imaging of transplanted beta cells or islet-like structures will likely be a valuable tool to readily assess function, immune response, and efficacy of matrices or other interventions geared to protect the transplanted material.

## Discussion and limitations

With beta cell dysfunction present in both T1D and T2D, a need exists to study the pathophysiology of beta cell failure. One emerging molecular technique to address this need is human islet transplantation into rodents coupled with intravital microscopy, an imaging approach to study the tissues of live animals in real time. Utilizing virally packaged biosensor technology, pre-transplant transduction of engraftable tissue allows processes of interest to be monitored *via* changes in spectral emission or intensity following treatment. More dynamic processes of sub-cellular function such as ion signaling, autophagic flux, and ROS production, among others, can therefore be quantitatively evaluated. Although virally packaged biosensors provide useful insight on cellular processes, it is important to note that biosensor expression is not as stable as with generation of a transgenic mouse line. However, when working with human islets, this genetically encoded form of biosensor expression is not a possibility. Additionally, during imaging, fluorescently conjugated probes and antibodies can also be injected to allow for further analysis in areas of interest like vascularity, immune infiltration, and cellular stress response in the beta cell. It is important to note that although fluorescent probes and antibodies are easier to acquire due to higher availability, these approaches do not allow for as much information on dynamic processes due to time required to reach targets and limited capabilities to combine fluorophores on the same probe or antibody. The ability to inject various treatments and study impact on beta cell function in real time enables physiologically relevant evaluation of novel drugs in the preclinical setting.

Along with the many benefits to using intravital microscopy as a tool to study beta cells *in vivo*, there are also many limitations when studying transplanted islets that have yet to be addressed. One major limitation of islet transplantation is the inability to transplant back into the endogenous environment due to the release of digestive enzymes from penetrating the pancreas. Many studies have shown that there is important crosstalk between the endocrine and exocrine tissues of the pancreas (95), and this communication is lost following transplantation. Furthermore, several transplant sites, such as the pinna of the ear and the subcutaneous space, require additional modulation to promote vascularization and successful engraftment. In mouse islet transplantation studies, reinnervation seems to never reach physiological levels, with studies showing that six weeks following transplantation, density of sympathetic nerves are only 60% that of endogenous islets in the pancreas (5). Together, this suggests that future studies need to address limitations with islet transplantation before intravital imaging may be performed to its peak potential.

The mouse models used for islet transplantation studies are also a limitation themselves, with most transplantation studies being performed in immunocompromised animals. Since type 1 diabetes is an autoimmune disorder, an important aspect of studying the disease in a relevant manner is diminished with transplantation of human islets into immunodeficient mice. With the development of humanized mouse models for studying immune interaction with transplanted islets, these concerns are somewhat ameliorated. However, continued development of these models to allow for robust evaluation of iPSC-derived beta cell clusters in the context of immune cells from the same donor will be critical to push the field forward.

Although this literary review has focused on the technique of performing intravital microscopy on transplanted human islets, it is important to note that other methods of non-invasive *in vivo* imaging have been performed. For example, researchers have conducted bioluminescence imaging, captured with IVIS imaging systems, on transplanted human islets expressing adenoviral luciferase under the cytomegalovirus (CMV) promoter, showing changes in islet mass following transplantation (96). To observe beta cell mass with higher accuracy following transplantation, researchers have employed the use of positron emission tomography (PET) imaging to study these changes with glucagon-like peptide agonist Exendin-4 *in vivo* (97). With PET imaging being a safe and efficacious way to monitor processes within the islet *in vivo*, this method has been used clinically to study endogenous changes in beta cell function within human subjects with T1D (98). Although magnetic resonance imaging (MRI) is an appealing clinically relevant model to image physiological processes *in vivo*, few MRI studies have been performed on transplanted human islets since the method was first introduced in 2011 (99). This lack of current research is due to challenges associated with generation of radiolabels, however, with the increased development of targeted nanoparticles in the field (100–102), there is a promising future for this imaging method (103). Although these discussed imaging studies have made important contributions to the field of diabetes with studies in islet mass following transplantation, these methods of non-invasive imaging lack the ability to acquire high-resolution images, losing the potential to visualize detailed dynamic processes occurring within the islets in real time.

In summary, though not without drawbacks, human islet transplantation into rodent models represents an exciting approach to study human islets in a physiologically relevant manner that currently cannot be recapitulated by *in vitro* approaches. When coupled to high-resolution imaging techniques such as intravital microscopy, these models represent an approach that has the potential to revolutionize our understanding of both basic islet biology as well as mechanisms of diabetes pathogenesis. Future development of both the models/approaches for human islet transplants in this context and the technologies to functionally evaluate the transplanted islets will undoubtedly revolutionize our ability to develop novel therapies to not only treat but also to prevent islet dysfunction and immune attack.

## Author contributions

This manuscript was written and edited by LW, OM, BD, and AL. All authors contributed to the article and approved the submitted version.

## Funding

Research involving human islet transplants into mice for intravital imaging in the Linnemann Lab is supported in part by grants from the National Institutes of Health, R03 DK 127766 and R01 DK 124380 to AKL and using resources and/or funding provided by the NIDDK-supported Human Islet Research Network (HIRN, RRID : SCR_014393; https://hirnetwork.org) including a HIRN New Investigator Award to AKL. 

## Acknowledgments

The authors would like to thank Dr. Donalyn Scheuner for helpful feedback and editing of this manuscript.

## Conflict of interest

The authors declare that the research was conducted in the absence of any commercial or financial relationships that could be construed as a potential conflict of interest.

## Publisher’s note

All claims expressed in this article are solely those of the authors and do not necessarily represent those of their affiliated organizations, or those of the publisher, the editors and the reviewers. Any product that may be evaluated in this article, or claim that may be made by its manufacturer, is not guaranteed or endorsed by the publisher.
